# Biomarkers of Oxidative Stress Tethered to Cardiovascular Diseases

**DOI:** 10.1155/2022/9154295

**Published:** 2022-06-24

**Authors:** Poojarani Panda, Henu Kumar Verma, Saikrishna Lakkakula, Neha Merchant, Fairrul Kadir, Shamsur Rahman, Mohammad Saffree Jeffree, Bhaskar V. K. S. Lakkakula, Pasupuleti Visweswara Rao

**Affiliations:** ^1^Department of Zoology, Guru Ghasidas Vishwavidyalaya, Bilaspur, India; ^2^Department of Immunopathology, Institute of Lung Biology and Disease, Helmholtz Zentrum, 85764 Neuherberg, Munich, Germany; ^3^Department of Public health, Nellore Municipal Corporation, Nellore, India; ^4^Department of Bioscience and Biotechnology, Banasthali Vidyapith, Banasthali, 304022 Rajasthan, India; ^5^Department of Emergency Medicine, Faculty of Medicine and Health Sciences, Universiti Malaysia Sabah, Kota Kinabalu 88400, Malaysia; ^6^Department of Biomedical Sciences, Faculty of Medicine and Health Sciences, Universiti Malaysia Sabah, Kota Kinabalu 88400, Malaysia; ^7^Department of Community and Family Medicine, Faculty of Medicine and Health Sciences, Universiti Malaysia Sabah, Kota Kinabalu, 88400 Sabah, Malaysia; ^8^Department of Biochemistry, Faculty of Medicine and Health Sciences, Abdurrab University, Jalan Riau Ujung No. 73, Pekanbaru, 28292 Riau, Indonesia; ^9^Centre for International Relations and Research Collaborations, Reva University, Rukmini Knowledge Park, Kattigenahalli, Yelahanka, Bangalore, 560064 Karnataka, India

## Abstract

Cardiovascular disease (CVD) is a broad term that incorporated a group of conditions that affect the blood vessels and the heart. CVD is a foremost cause of fatalities around the world. Multiple pathophysiological mechanisms are involved in CVD; however, oxidative stress plays a vital role in generating reactive oxygen species (ROS). Oxidative stress occurs when the concentration of oxidants exceeds the potency of antioxidants within the body while producing reactive nitrogen species (RNS). ROS generated by oxidative stress disrupts cell signaling, DNA damage, lipids, and proteins, thereby resulting in inflammation and apoptosis. Mitochondria is the primary source of ROS production within cells. Increased ROS production reduces nitric oxide (NO) bioavailability, which elevates vasoconstriction within the arteries and contributes to the development of hypertension. ROS production has also been linked to the development of atherosclerotic plaque. Antioxidants can decrease oxidative stress in the body; however, various therapeutic drugs have been designed to treat oxidative stress damage due to CVD. The present review provides a detailed narrative of the oxidative stress and ROS generation with a primary focus on the oxidative stress biomarker and its association with CVD. We have also discussed the complex relationship between inflammation and endothelial dysfunction in CVD as well as oxidative stress-induced obesity in CVD. Finally, we discussed the role of antioxidants in reducing oxidative stress in CVD.

## 1. Introduction

Cardiovascular diseases (CVDs) are a group of ailments that affect the heart and the blood vessels. Primary types of CVDs include coronary heart disease, rheumatic heart disease, peripheral arterial disease, cerebrovascular disease, and stroke. According to the World Health Organization (WHO), CVD is a complex disease and a leading cause of death worldwide accounting for 31% of all fatalities. CVD is mainly caused by the deposition of fatty acids in the inner wall of the blood vessels. Approximately, 17.6 million people have died due to CVD in 2016. According to a report from the Royal College of the cardiologist, the number of CVD-related facilities increased by 14.5 percent in the United Kingdom between 2006 and 2016 [1]. Several factors contribute to CVD such as high cholesterol, smoking, and elevated blood pressure but a major contributing factor still remains oxidative stress. Oxidative stress generates free radicals and reactive oxygen species (ROS); when these are abundant, the primary structures that are affected in cells are DNA, lipids, and proteins, resulting in cell death [2]. The processes such as neurological system deregulation, immune cell degeneration, and response control are known to be affected by ROS exhibiting both positive and negative effects. Oxidative damage to the cell in CVD causes myocyte dysfunction, which leads to cell death [3]. ROS affect contractile function directly by altering the proteins that are involved in excitation-contraction coupling. Therefore, reduced ROS formation should be employed to prevent and treat cardiovascular disease [4]. However, analyzing redox systems is difficult due to significant subcellular variations in redox potential and the short lifetime of ROS. The discovery of different biomarkers of oxidative stress warrants further investigation to evaluate CVD [5]. Despite significant efforts, elucidating the pathophysiologic pathways driving the onset and progression of CVD still remains a work in progress [6–8].

The purpose of this review is to demonstrate the relationship between oxidative stress and CVD with emphasis on the following points: oxidative enzymes cause endothelial dysfunction, oxidative stress biomarkers that are involved in CVD, the link between oxidative stress-induced inflammation and CVD, and the vital role of different antioxidants in lowering free radical levels in CVD.

## 2. Oxidative Stress and ROS

Oxidative stress is characterized as a natural imbalance between the production and accumulation of ROS, which plays a role in the ageing process [9, 10]. ROS are oxidants and an excessive ROS induces oxidative stress. ROS generation and the intracellular immune system are important regulators of intracellular oxidative equilibrium. Increased cellular ROS production plays a vital role in LDL oxidation, endothelial dysfunction, and inflammatory processes [11]. Excessive ROS production exhibits a negative impact on the biological system by serving as a second messenger in cellular signaling and affecting natural components like lipids, proteins, and DNA [12]. Various environmental elements, such as UV rays, radiation, smoking cigarettes, and heavy alcohol use, encourage ROS generation that leads to the emergence of several illnesses like cancer and CVD [13–15].

## 3. CVD Concerning Oxidative Stress

According to the WHO, CVDs are complex diseases that cause a significant number of deaths worldwide. An increase in ROS production has been linked to various cardiovascular disorders [16–19]. Oxidative stress has been strongly linked to myocardial infarction (MI), ischemia/reperfusion, and heart failure [20]. Heart attacks and strokes are unexpected occurrences that are caused due to the lack of blood supply to reach the heart or brain as a result og blockage. Atherosclerosis is the most prevalent cause of CVD. It is caused by artery hardness and constriction, which results in decreased oxygen and blood distribution throughout the body [21]. Plaque development in the inner walls of the coronary artery is characterized by the deposition of large amounts of LDL cholesterol, cellular debris, and other elements [22]. Lipids, especially polyunsaturated fatty acids (PUFAs) and cholesterol, are critical oxidative stress target substrates as lipids that make up cell membranes. Various reactive aldehydes are generated during lipid oxidation depending on the type of PUFA trans-4-hydroxy-2-nominal, isoprostanes, and malondialdehyde [23]. Within macrophages, oxidized LDLs are endocytosed, which is a gradual process that leads to a build-up in the intima and culminats in the atherosclerosis. Studies on endothelial cell damage produced by LDL modifiers have shown that glycated LDL (gLDL) and oxidized LDL (oxLDL) are associated with atherogenic processes. Modified natural LDL components such as apo*β*, a surface protein of LDL, improve the ability of LDL to attach to its receptor [24]. In contractile failure, the contractility is altered by the oxidation of Sarco and endoplasmic Ca+2-ATPase as well as contractile proteins, including tropomyosin and actin [25, 26]. The impact of ROS on endothelium underpinning molecules that can induce death, inflammation, and therefore clotting plaque formation in atherosclerosis makes oxidative stress a critical characteristic of CVD, which is also characterized as an initial causal component [27, 28].

## 4. Sources of ROS in CVD

**Figure 1:**
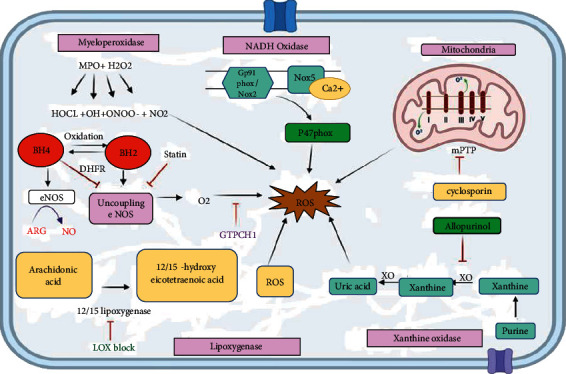
Schematic representation and molecular mechanism of the sources of ROS generation in CVD. Mitochondria is the leading cause of ROS generation; complexes I and III produce superoxides and open mPTP pores in the mitochondrial membrane through ROS release. NOX2/gp91phox generates O^2^^•^, and other members of the NOX1, NOX4, and NOX5 family are known to be involved in ROS generation. Xanthine oxidase accepts an electron from O^2^ and produces O^2•^ while its action can be blocked by allopurinol. Lipoxygenase produces ROS by acting on arachidonic acid into HETE, and LOXBlock-1 blocks this step. When eNOS is uncoupled, it forms O_2_^•^ instead of NO and helps in ROS production. MPO is the critical oxidative stress biomarker that reacts with H_2_O_2_ and produces various super radicals, which are the primary source of ROS.

Both endogenous and exogenous factors produce ROS in CVD. Mitochondria are the primary source of endogenous ROS via the electron transport chain (ETC) and oxidative phosphorylation that produce by-products in CVD [29]. Mitochondria is the primary source of ATP synthesis involving complexes I to V. The mitochondria's respiratory chain is disturbed during pathological conditions. Oxygen drains into the electron to form superoxide [30]. Isoproterenol or Ang II leads to increased mitochondrial superoxide anion generation during cardiomyocyte hypertrophy [31, 32]. Increased oxidative stress and ROS generation are linked to mitochondrial respiratory chain abnormalities; as a result, many transcription factors and protein kinase signaling pathways that are involved in cardiac hypertrophic are activated [33]. Mitochondria is the primary generator of ROS, especially in cardiac cells [27]. The two most prevalent ROS generating sources in the ETC are complex I as well as complex III to facilitate pore opening (KATP and mPTP) in mitochondrial outer and inner membranes [34]. Cyclosporin is the inhibitor of mPTP [35]. In a recent study, complex II ROS possesses cardioprotective properties, whereas complex I ROS generation causes damage and is linked to CVD [36]. Complex III-produced ROS causes delayed channel opening and is associated with improved functional recovery following injury to the ischemic-reperfusion, which is identified as a major risk factor of oxidative stress [37]. Xanthine oxidase, lipoxygenase, myeloperoxidase (MPO), noncoupling nitric oxide synthase (NOS), and NADPH oxidase (NOX) are known to be associated with ROS generation [38] (Figure 1). Several investigations have revealed that the mitochondria and NOX are both significant ROS producers and hyperglycemia is linked to mitochondria-NOX ROS interaction [39]. Further, mitochondrial biogenesis and mitophagy pathways that regulate mitochondrial dynamics are critical in maintaining healthy cardiomyocytes [40]. Ketogenic diet can accelerate cardiac fibrosis by reducing mitochondria-associated membranes and inhibiting mitochondrial function [41]. Secreted frizzled-related protein 2 (SFRP2) that interfere with Wnt is known to modulate mitochondrial dynamics and mitochondrial biogenesis as well as exert cardioprotective effects within diabetic cardiomyopathy [42].

### 4.1. NADPH Oxidase (NOX)

NADPH oxidase is the major enzyme involved in ROS production within the mitochondria. NADPH oxidase produced in mammalian phagocytes is the first known enzyme specialized to generate O_2_ [43]. NOX uses NADPH or NADH as an electron donor to catalyze the conversion of dioxygen into superoxide anion. NOX family members responsible for the generation of ROS in CVD are NOX1, NOX2, NOX4, and NOX5. The oxygen synthesis mainly characterizes NOX enzymes; however, NOX4 is an outlier due to its primary hydrogen peroxide production (H_2_O_2_) [21]. NOX2 and NOX4 were shown to generate ROS in cardiomyocytes and fibroblasts, whereas NOX1, NOX4, and NOX5 were identified in vascular smooth muscle cells [44]. The control of NOX2, also called gp91phox, is being extensively researched. NOX2 is activated by other NOX subunits such as p22phox, p67phox, p40phox, and p47phox. p47phox activation is caused by the phosphorylation at Ser303, Ser304, and Ser328. The stimulated p47phox then interacts with p22phox, allowing NOX2 to access p40phox and p67phox, culminating in NOX2 activation [45]. Upon NOX2 activation, NADPH can bind to intracellular C- terminus, leading to ROS on the extracellular membrane. Furthermore, secondary oxidase systems such as NOS uncoupling, mitochondrial malfunction, and XO activation are stimulated by NOX-derived ROS [12, 46]. NOX5 is calcium dependent and controlled by calcium stimuli; its involvement in CVD is considerably more complicated. NOX4 remains active within the cell in the presence of O_2_ [47].

NOX4 has been found to enhance ROS production upon TGF-*β* stimulation [48]. NOX4 regulates smooth muscle-actin upon being expressed by inactivating p38MAPK through MKP-1 oxidization. Moreover, NOX4 activates the RhoA protein kinase, which activates the serum response factor (SRF) and controls the level of myocardin-related transcription factor (MRTF). Subsequently, this leads to the activation of spinal muscular atrophy (SMA). As a result, when NOX4-ROS inhibits the activation of MKP-1 and p38MAPK, p38MAPK phosphorylates SRF, thereby causing VSMC differentiation that is linked to vascular damage [21]. Several lines of evidences indicate that the prediabetic patients with mildly elevated glucose levels are found to be linked with increased risk of heart failure [49, 50] and worse prognosis in patients with heart failure (HF) [51]. High glucose levels have been linked to increased NOX4 synthesis in cardiomyocytes, contributing to heart injury. Overexpression of NOX4 has also found to lower baseline blood pressure, which is connected to H_2_O_2_ generation [52].

NOX2 and NOX4 levels are elevated in pulmonary artery hypertension (PAH), and its blockage has shown to revere PAH in animal models [53]. Neutrophil-NOX synthesis is a potent antibacterial agent, which is identified to reduce molecular oxygen into O_2_, dismutation into H_2_O_2_, NOX 4, and chloride ion in hypochlorous acid [54]. Further, upregulation of NOX2, NOX4, and NOX5 has been linked to CVD and atherosclerosis during the early stages of the disease [21].

### 4.2. Xanthin Oxidase (XO)

The dehydrogenase form of XOs accepts electrons from both molecular oxygen and NAD^+^, whereas the oxidase form of XOs accepts electrons solely from the molecular oxygen [55]. XO is the first biological process recognized as a source of ROS. Endothelial dysfunction has been related to the generation of XO-ROS, which is caused by the interaction of O_2_ and NO, resulting in the formation of OONO^−^ that causes cellular damage. In CVD patients, hyperuricemia or elevated uric acid levels are a common occurrence. Xanthine oxidoreductase is an enzyme that is responsible for the synthesis of uric acid and plays an important role in the pathophysiology of CVD [21]. Elevated uric acid is a biomarker for fatal heart failure; hence, XO inhibition will assist in positively treating patients with heart defects [56]. NOX activity is required for Ang II in order to activate XO, as Ang II-induced superoxide generation could be prevented by NOX inhibition [55]. Endothelial XO activity has been shown to be elevated in individuals with coronary artery disease. Allopurinol is an XO inhibitor that may benefit HF patients with endothelial dysfunction [57].

Furthermore, XO's product can be used as a biomarker to detect cardiovascular disorders [58]. Pulmonary hypertension has been linked to reduced XO activity through the early growth response-1 (Egr-1) signaling pathway [59]. Erg-1 expression is induced by XO-ROS, which ultimately leads to CVD [60].

### 4.3. Lipoxygenase

Lipoxygenase (LOX) oxidizes arachidonic acid (AA), a polyunsaturated fatty acid produced by the plasma membrane following phosphatidylcholine hydrolysis, in order to produce hydroperoxides. 12-LOX and 15-LOX convert arachidonic acid to 12- and 15-hydroxyeicosatetraenoic acid, respectively, and generate ROS (ROS). LOXBlock-1 is the inhibitor of 12/15 lipoxygenase [61]. Hyperglycemia activation of 12/15-LOX is linked to increased cardiovascular oxidative stress and diabetic cardiomyopathy [39]. In rat fibroblasts, cPLA2-arachidonic acid-related cascade might lead to a rise in ROS production via the lipoxygenase pathway. Metabolism of AA with the help of 5-lipoxygenase and cPLA2 activation was shown to be involved in the production of ROS induced by tumor necrosis factor-*α* (TNF-*α*) [62]. NSAIDs induce leukotrienes and 5-lipoxygenase levels in the body and increase ROS generation [62]. Through LOX, Ang II causes NOX to be produced in VSMCs and AA catalysis results in ROS being produced in vascular cells. 5-LOX is elevated by cytotoxicity and oxidative stress (NOX or mitochondrial ROS), while 5-LOX catalysis of AA includes leukotriene (LTA4) and 5-HETE lipid mediators [63]. Several proinflammatory molecules are produced by LTA4 metabolism including LTB4, LTC4, LTD4, and LTE4 that subsequently connect with and activate other cells like endothelial cells, neutrophils, mast cells, macrophages, foam cells, and T-cell. Upon activation, these cells release cytokines and metalloproteinase, which exhibit proatherosclerotic functions [64]. LDL molecule oxygenation is a defining feature of 12/15-lipoxygenase action. In diabetic heart patients, inhibition of 12/15-LO leads to a decrease in 4-hydroxy-2-nonenol (4-HEN), an oxidative stress marker, ROS generation, and NOX4 related to 12/15-LO activity [65].

## 5. Noncoupling Nitric Oxide Synthase

Nitric oxide synthase (NOS) occurs in three forms: inducible NOS (iNOS), endothelial NOS (eNOS), and neuronal NOS. NOS activity produces NO in the cardiovascular system (nNOS) [56]. NO has been included in several clinical studies including CVD, diabetes, and hypersensitive stress, in order to support vasodilation and enzyme failure linked to NO generation. Endothelial nitric oxide synthase may produce NO from l-arginine. BH4 deficiency due to vascular homeostasis regulation caused by eNOS leads to the production of free radicals [62]. When exposed to oxidative stress, BH4 is transformed to 7,8-dihydrobiopterin (BH2), which can bind to eNOS, induced uncoupling, and resulted in the production of O_2_^•^ rather than NO, which causes oxidative stress to develop [66].

Upregulation of eNOS has been proven to protect against heart dysfunction. It has been shown that during coronary artery ligation, better left ventricle function and decreased left ventricle hypertrophy in transgenic mice are increased by 30-fold during cardiomyocyte eNOS activity [67]. In contrast, cardiomyocyte-specific NOS mutant mice were reported to have dramatically improved survival following heart failure caused by MI, suggesting that iNOS may be implicated in the etiology of heart failure [68].

•NO interacts with molecular oxygen and ROS (ROS) to produce various oxidation products, including RNS. The interaction of •NO with superoxide (O2•) can make peroxynitrite (ONOO−) at virtually diffusion-limited rates, one of the most significant RNS-generating reactions. It also acts as an oxidant when protonated, undergoing homolytic scission to produce nitrogen dioxide and hydroxyl radical (•OH) [69].

## 6. Myeloperoxidase (MPO)

Myeloperoxidase is a member of the heme peroxidase superfamily [70]. MPO is implicated in both inflammatory and oxidative stress pathways in CVD development. MPO is found in large amounts in neutrophils and at lower levels in monocytes. Neutrophils also produce superoxide, which can oxidize other molecules or transform into hydrogen peroxide by SOD. MPO employs hydrogen peroxide to generate several potent oxides, including hypochlorous acid (HOCL), hydroxyl radicals (OH), nitrogen dioxide (NO_2_), and peroxynitrite (ONOO) [71]. MPO produces ROS, which can alter lipids, lipoproteins, and proteins. SCN is produced in large quantities by smoking and its interaction with hydrogen peroxide (H_2_O_2_). MPO causes considerably an elevated hypothyocianous acid (HOSCN) level [72]. HOSCN may also alter LDL and HDL to OXLDL and OXHDL as well as oxidize NO, resulting in cyanate, which interacts with amides to produce a CVD prediction marker [21]. Recent research has found a connection between IR with increased MPO production in leucocytes and enhanced ROS formation in polycystic ovary syndrome (PCOS) patients, emphasizing MPO's role in oxidative stress events [73].

## 7. Endothelial Dysfunction in CVD

Endothelial dysfunction is a proinflammatory and prothrombotic condition characterized by the production of cell surface adhesion molecules that are required for the recruitment and attachment of inflammatory cells [74, 75]. Endothelial dysfunction is linked to oxidative stress and inflammatory processes. All these factors are all involved in the etiology of cardiovascular mortality and morbidity [76, 77]. Endothelial cells have a vascular disease-fighting enzyme called endothelial nitric oxide synthase (eNOS) [78]. This chemical diffuses vascular smooth muscle cells, while activating the cGMP pathway [79]. Nitric oxide (NO) is the most critical factor in maintaining vascular homeostasis in endothelial cells [80]. Endothelial dysfunction is marked by decreased NO bioavailability due to decreased NO synthesis or NO breakdown by superoxide anion. When the superoxide anion interacts with NO, peroxynitrite ONOO^−^ is produce [81]. The inactivation of NO by various oxidative enzyme systems is a critical process that leads to endothelial dysfunction via an increase in superoxide anion levels [81]. NADPH oxidase and eNOS uncoupling are the primary sources of O_2_ generation that gives rise to vascular oxidative stress [82].

Several investigations have found that oxidative stress influences cytokine synthesis and release [83–85]. Increased levels of proinflammatory cytokines such as tumor necrosis factor (TNF), interferon-gamma, and interleukin 1 (IL-1) have been linked to aging-related endothelial dysfunction [86]. Inflammatory cytokines, ROS, lipid, and mechanical forces acting on the endothelium wall of blood vessels can activate the NF-*κ*B pathway [5]. TNF-*α*-induced CD40 expression has been shown to change the certain adhesion molecules' level of expression and increase the inflammatory reaction [87]. Resveratrol therapy reduces CD4 expression; it also suppresses the generation of ROS triggered by TNF-*α* via stimulating the activity of sirtuin-1, a histone deacetylase associated with inflammation suppression and protecting cells from damage caused by inflammatory stimuli [88].

Apelin is a peptide encoded by the APLN gene in humans. Apelin is one of two endogenous ligands for the G-protein-coupled APJ receptor found on the surface cells. Many studies have found that apelin plays a vital role in the endothelial cells and is well known for its impact on cardiac myocytes [89–91]. Apelin increases NO bioavailability in endothelial cells [92]. Apelin is an anti-inflammatory drug as it inhibits the synthesis of inflammatory mediators, including interleukin-1 and TNF-*α* [93]. Apelin protects against oxidative stress and reduces I/R in cardiac cells by inhibiting mitochondrial oxidative stress damage and peroxidation of lipids [94]. Therefore, an increase in the oxidative stress in apelin-deficient heart tissues causes decreased NO bioavailability and activation of the inflammatory protein, which ultimately leads to endothelial dysfunction.

**Figure 2:**
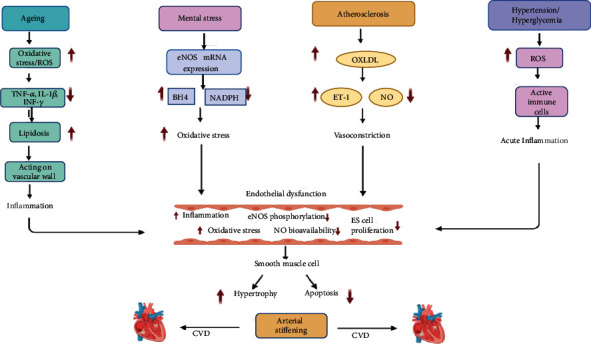
Schematic representation of the primary mechanisms involved in endothelial dysfunction in CVD. Endothelial cell rich in eNOS enzyme that produces NO is an essential compound in the endothelial function. eNOS acts on the cGMP pathway in VSMC and causes vasodilation. When eNOS is uncoupled, it reduces NO's production, which leads to decreased vasodilation properties and causes endothelial dysfunction. Oxidative stress is enhanced during aging, elevating the release of cytokines and ROS production; these act on the vascular wall and cause inflammation. Mental stress in CVD reduces eNOS mRNA expression and decreases BH4 activity, enhancing ROS generation and reducing NO bioavailability. OXLDL levels are high during atherosclerosis and increase the release of the ET-1 vasoconstrictor, which is also a significant factor of endothelial dysfunction. Other CVD risk factors like smoking, hypertension, and hypercholesterolemia generate ROS, causing inflammation and ultimately leads to endothelial dysfunction.

Endothelial dysfunction caused by mental stress is an initial cause of developing severe CVD [95]. Glucocorticoid and endothelin-1 (ET-1) play a role in causing stress [81]. Extreme stress reduces eNOS mRNA and protein expression and increases the ROS level by depleting BH_4_ and activating NADPH oxidase. OxLDL, a cardiovascular risk factor, increases endothelin-1 synthesis [96]. ET-1 suppresses the vasodilating effects of NO by activating ETA receptors and exerting a vasoconstrictive effect. Endothelial dysfunction indicators include lipid peroxide, nitrotyrosine, and NO [97] (Figure 2).

## 8. Biomarkers of Oxidative Stress in CVD

The National Institute of Health defines a biomarker as “an objectively measured and assessed feature that serves as an indication of normal biological process, pathogenic process or pharmacological reactions to a therapeutic intervention.” The evidence for directing therapy and improving patient outcomes defines a biomarker's clinical usefulness [98]. Biomarkers that closely correspond to the disease pathophysiological pathway are the most favorable ones. ROS has been quantified as a potential biomarker for disease progression because of its significant role in oxidative stress in CVD pathogenesis [99]. Molecules that are altered during its interaction with ROS and the antioxidant system molecules that vary in response to increasing redox stress are the two diverse biomarkers for oxidative stress [100]. Over 20 distinct markers have been discovered in meta-analyses of diseases like depression; however, the number is likely to be greater, making it impossible to draw significant conclusions [101].

To date, several vital biomarkers of CVD oxidative stress have been identified. Since the oxidative hypothesis of atherogenesis has been proposed, the importance of free radical oxidation of cellular components in CVD has been recognized [102]. Lipid peroxidation (isoprostane and malondialdehyde), oxidative protein modification (nitrotyrosine, S-glutathionylation), myeloperoxidase, oxidized low-density lipoprotein, and oxidized lipoprotein are the sources of oxidative stress biomarkers. Detailed biomarkers and pathways are depicted in Table 1.

### 8.1. Lipid Peroxidation

Numerous plasma markers of lipid peroxidation are available, such as malondialdehyde (MDA), lipid hydroperoxides, oxysterols, and F2-*α* isoprostanes [71]. Isoprostanes and MDA have widely researched indicators for lipid peroxidation. Isoprostanes and MDA are formed by the peroxidation of polyunsaturated fatty acids that are found in the phospholipids of the cell membrane [103]. Further, phospholipids release isoprostane from the cell membrane that is measured in tissue, blood, and urine [104]. Its concentration is determined using gas chromatography-mass spectrometry (GC/MS), ELISA, liquid chromatography-mass spectrometry (LC/MS), urine sample, and radio immune assay in plasma [105]. MDA can be measured using a colorimetric assay, an ELISA, an HPLC, or a TBRS assay. Different antioxidants like lipoic acid and aminoguanidine can adjust plasma TBRS concentration [106].

### 8.2. Protein Oxidation

Protein oxidation can be triggered selectively by ROS (ROS) and nitrogen species (RNS) in multiple ways [107]. Nitrotyrosine and S-glutathionylation are the two most essential biomarkers of CVD. The reaction of protein tyrosine nitrate is mediated by reactive nitrogen species such as peroxynitrite (ONOO) and nitrogen dioxide (NO_2_) with its transition metalcore, MPO that can respond with ONOO to produce the oxo metal complex and NO_2_ to facilitate the nitrogen response [103]. The current gold standard method for quantifying free nitrotyrosine (3-NO2-TYR) is tandem mass spectrometry (MS/MS) combined with GC or HPLC. The immune cytochemical and histochemical assays are based on monoclonal or polyclonal anti-3-NO2-TYR antibodies [108]. The level of plasma protein-bound nitrotyrosine was very significant for CVD patients, even after adjusting conventional CVD and CR risk factors [109]. Trimethylamine N-oxide (TMAO) is a tiny amine oxide produced by gut microbial metabolism. The elevated plasma TMAO level in patients with HF is associated with poor prognoses [110].

S-Glutathionylation creates a disulfide bridge between the cellular tripeptide glutathione and reactive cysteine residue [111]. By this oxidative modification, the tertiary structure of protein gets changed and redox regulation of various activities including ryanodine receptor, eNOS; Na + k + pump etc. This impacts proteins and can alter the intracellular Na + −K + pump and the signaling pathway of CVD [112]. The level of S-glutathionylation of sensitive protein can be measured by Western blotting [113], ELISA with monoclonal anti-glutathione antibody, and MS technique [114].

### 8.3. OX-LDL and Oxidized Phospholipids

Low-density lipoprotein oxidation is possible nonenzymatically, or enzymes like 12/15 lipoxygenase can catalyze the LDL [115]. OXLDL can be quantified using monoclonal antibodies that detect unusual oxidation based on a specific epitope; currently, available plasma OXLDL ELISA techniques are OXLDL-EO6 ELISA assay (quantify oxidation of phospholipid on apo-B-100), LDL-DLH 3 (quantify apoB100 on oxidized phospholipids), and OXLDL-4E6-sandwich ELISA (for MAD-LDL and copper OXLDL detection). It has been observed that OXLDL levels are higher in CVD patients [103]. Multiple studies have indicated that the long-chain (LC) omega-3 polyunsaturated fatty acids (n-3 PUFAs) significantly decrease the risk of fatal coronary heart disease [116]. However, no association between *α*-linolenic acid (ALA) and the risk of HF was documented [117].

### 8.4. Myeloperoxidase (MPO)

MPO functions as a master heme enzyme that helps generate ROS by converting hydrogen peroxide (H_2_O_2_) into OH, ONOO, NO_2_, and HOCL. ROS generated by this process can alter lipid, protein, and lipoprotein [118]. The MPO level can be quantified by peroxidase assay, spectrophotometry, and ELISA. The presence of CAD was related to higher circulating MPO levels [119]. This leads to chest pain, increases the risk for MI, and can even be fatal. Because of this, MPO is the most effective biomarker of CVD [120].

## 9. Secreted Frizzled-Related Proteins (SFRPs)

Secreted frizzled-related proteins (SFRPs) are Wnt antagonists. By interfering with the Wnt signaling pathway, it downregulates axin-related protein 2 (AXIN2) and matrix metalloproteinase-7 (MMP7) proteins [121]. Sfrps family members influence cellular apoptosis, angiogenesis, differentiation, the inflammatory process, and cardiac remodelling [122]. SFRP2 can regulate cardiac development and CVD [123]. Further, SFRP2 remains an independent biomarker for myocardial fibrosis [124]. Measurement of secreted frizzled-related protein 5 (SFRP5) levels in HF patients with or without T2DM indicated that elevated SFRP5 levels decreased rehospitalization or all-cause mortality in HF patients with T2DM [125].

## 10. Clinical Significance of Biomarkers

Despite its physiopathological significance and promising preclinical findings, no redox biomarkers such as myeloperoxidase, protein and lipid oxidation, advanced glycation end products (AGEs), and their soluble receptor have made it to clinical practice. This could be attributed in part to a lack of data, the redox balance's complexity, and high level of interaction between these indicators and several established risk factors (age, renal failure, smoking, inflammatory disease, and drugs) [144].

## 11. Disadvantages of Biomarkers

The prevalence of a wide range of oxidative stress biomarkers does not imply superiority of any particular biomarker over the other; however, it is critical to compare the oxidative stress assessment's value with commonly used risk scores. This property must be considered when deciding the biomarker to be used in a specific situation [145]. The nitrotyrosine levels in the circulatory system differ from those in the tissues; besides, S-glutathionylation detection is susceptible to methodological artifact [103]. Subsequently, it has been revealed that MDA is not a specific biomarker as the TBARS assay can detect aldehydes except MDA [146].

### 11.1. Oxidative Stress, Obesity, and CVD

Obesity is a primary risk factor for metabolic and CVD. Oxidative stress is essential for obesity-related diseases, including dyslipidemia and hypertension in CVD [147]. Adipokines and other types of bioactive chemicals are released from the adipose tissue, and its adipokines include interleukin-6 (IL-6), TNF-*α*, monocyte chemo attachment protein-1 (MCP-1), and adiponectin [147, 148]. Obesity combined with dyslipidemia has been proven to enhance the onset of CVD associated with oxidative stress [149]. Low-density lipoprotein (VLDL) increases the production of ROS (ROS) in the endothelium, which can disrupt the lipid and protein signaling pathway [150]. ROS-mediated changes in lipids such as oxidized LDL (OX-LDL) and peroxidized lipoproteins either directly or indirectly stimulate adipocyte growth by increasing monocyte/macrophage infiltration [151]. Activating lipoprotein lipase causes fatty acid build-up in the adipocytes [152]. OX-LDL reduces the release of adiponectin from adipose tissue limiting ROS formation. Reduced adiponectin levels have been linked to coronary artery disease (CAD) [153].

An increase in ROS substantially impacts the biology of white adipose tissue, leading to unregulated production of inflammatory cytokines like tumor necrosis factor (TNF-*α*), thereby contributing to CVD [154]. Adiposite is associated with oxidative stress while mitochondria are the primary source of ROS. However, the mitochondrial action in the dysfunction of adipose tissue during obesity is a critical event in obesity. In this context, the elevated level of oxidative stress is a primary cause of CVD [155]. Adiponectin can reduce the production of adhesion molecules like intracellular adhesion molecule-1 by blocking TNF-mediated activation of nuclear factor kappa B (NF-*κ*B) endothelial cells. This results in the inhibition of monocyte adhesion, which is an early stage of atherosclerotic CVD [156]. Adiponectin decreases atherosclerosis, reducing pressure overload-induced myocardial hypertrophy and angiotensin-II-induced cardiac fibrosis, and protects the heart from ischemia [157]. Further, oxidative stress activates SNS in the brain that plays a significant role in the pathogenesis of obesity-related hypertension [158].

### 11.2. Antioxidants in CVD

Antioxidants are chemicals that protect the damaged cellular components via oxidative stress chain reaction [159]. Antioxidants limit ROS production by eliminating free radicals and enhancing cellular structures like lipid, protein, and DNA damage by ROS [160]. Antioxidants can be classified into enzymatic and nonenzymatic antioxidants. Examples of enzymatic antioxidants are superoxide dismutase (SOD), glutathione reductase (GRX), glutathione peroxidase (GPX), and catalase (CAT). Nonenzymatic antioxidants include endogenous antioxidants like glutathione (GSH), uric acid, albumin, and exogenous antioxidant like ascorbic acid (Vit-C), carotenoids, flavonoid, and vitamin-E [161].

#### 11.2.1. SOD

Superoxide dismutase is catalyzed by SOD and converted to H_2_O_2_ and O_2_. Catalase is an enzyme that adds in the conversion of H_2_O_2_ into H_2_O and O_2_ [162]. SOD has been proven to slow the progression of atherosclerosis [163].

#### 11.2.2. GPX

Red cell GPX isoform-1 exhibits significance in patients with coronary artery disease in addition to conventional risk factors [164]. The paraoxonase enzyme is linked to high-density lipoprotein (HDL). They have peroxide-like activity and protect lipoprotein against oxidation [165].

#### 11.2.3. TRX

TRX can scavenge ROS (ROS) such as H_2_O_2_ and ONOO^−^ [164], while oxidative damage is elevated upon TRX inhibition. TRX has reduced disulfide component, which is increased under oxidative stress. A study suggests that fibrosis, oxidative stress, and apoptosis are reduced in cardiomyocytes and endothelial cells as compared to the diabetic group with MI, a group lacking TRX1 overexpression [166].

#### 11.2.4. Vitamin E

The role of vitamin E supplementation in preventing CVD is controversial. When vitamin E was administered alone, MI was significantly reduced [167]. Vitamin E and vitamin C act as an inhibitor for LDL oxidation by reducing ROS formation and increasing NO bioavailability [168].

#### 11.2.5. Vitamin C

Vitamin C is an electron donor (reducer) with an antioxidant effect due to its ability to reduce oxidized species or oxidant radicals. Through various mechanisms, vitamin C contributes to the overall pool of nitric oxide (NO) and increases the bioavailability of NO. Vitamin C neutralizes superoxide radicals, stabilizes BH4, increases eNOS activity, and maintains L-arginine and cGMP levels [169]. It partly mediates antiapoptotic and antioxidant functions and exerts protective effects on human umbilical vein endothelial cells through the regulation of miRNA/mRNA axis expression [170]. Growing evidence also shows that H_2_O_2_-mediated endothelial cell senescence may be associated with abnormally expressed Piwi-interacting RNAs (piRNAs) and PIWI proteins. Furthermore, vitamin C is known to attenuate H_2_O_2_-mediated abnormal expression of Piwi-interacting RNAs (piRNAs) [171].

#### 11.2.6. Vitamin A

Vitamin A has been found to influence apolipoprotein on MI risk [29].

#### 11.2.7. Nuts

Nuts are known to lower the risk of CVD as it can inhibit lipid peroxidation and LDL oxidation in LDL particles [5, 172]. The EURAMIC trial discovered a minor link between the adipose-carotene content and the MI risk; however, the effect was lost after accounting for several confounding variables. Findings from a recent trial including 1031 Finnish males revealed a vital link between serum-carotene and a lower risk of MI [173]. Statin, *α*-tocopherol, and ascorbic acid have been used to prevent CVDs which are also antioxidant drugs [161]. It has been claimed that using antioxidants indiscriminately might be harmful. The quantity of antioxidants and oxidative stress should be in a careful equilibrium in our cells, which is crucial for optimal cell function. Disrupting this equilibrium can have unfavorable consequences. Furthermore, natural antioxidant defenses may be considerably more essential than antioxidants obtained from food or supplements to humans [174]. The use of antioxidant supplements as a treatment for oxidative stress is still debatable. However, it should be emphasized that antioxidants cannot repair damage in CVD patients but can only control disease consequences.

## 12. Conclusion and Future Perspective

Elevated oxidative stress in the body may lead to various pathophysiological conditions such as CVD. Involvement of free radicals like ROS and NOS affects the cardiovascular system causing multiple cardiac disorders. Some superoxide causes endothelial dysfunction of vascular tissue by reducing the bioavailability of NO. Oxidative stress causes cytokine secretion and promotes inflammation in the vascular tissue. Adhesion molecules act on the endothelial blood vessel wall and are the major cause of atherosclerotic plaque formation. Several oxidative stress biomarkers can detect CVD; however, these biomarkers are not enough to identify CVD and additional research is warranted. Antioxidants can reduce the effect of oxidative stress on CVDs. Many antioxidant drugs have been discovered so far that assist in decreasing the CVD mortality rate. There is an urgent need to identify and discover new methodologies and effective biomarkers that can be implemented to better understand the ROS mechanism towards CVD pathophysiology.

## Figures and Tables

**Table 1:** Characteristics of the included studies investigating the association between oxidative stress biomarkers in CVD.

Biomarkers	Study design	Pathway involvement	Laboratory methods	Model	Findings	Specificity	Reference
Isoprostane	Case-control	Peroxidation of polyunsaturated fatty acid catalyzed by free radical	GC/MS, ELISA, urine sample, radioimmune assay	In vivo, in vitro, ex vivo	F2-isoprostanes are predictive of peripheral and coronary artery disease	Nonspecific	[105, 126–128]
Malondialdehyde (MDA)	Cross-sectional	Peroxidation of polyunsaturated fatty acid, a side product of the thromboxane A_2_ pathway	Calorimetric assay, TBRS assay, ELISA, HPLC	In vivo	TRBAS blood serum levels in cardiovascular event	Specific	[103, 104, 109]
S-Glutathionylation	Case-control	Protein oxidation, glutathionylation pathway of protein	Western blotting, ELISA with monoclonal anti-glutathione antibody, MS	In vivo	It causes changes in intracellular Na+ and Ca2+ processing and other critical signaling pathways of CVD	Specific	[129–131]
Nitrotyrosine	Case-control	Tyrosine nitrate-mediated protein oxidation. ERK1/2 pathway	MS/MS, GC/MS, HPLC, immunocytochemical and immune histochemical assay based on monoclonal and polyclonal antibodies	In vivo	Nitrotyrosine enhanced fibrinogen activity and clot formation speed. Plasma protein-bound nitrotyrosine values are higher in coronary artery disease	Specific	[132–135]
OX-LDL	Nested case-control, cohort study	Autophagy-lysosome pathway, lipoxygenase-catalyzed oxidation of LDL	Monoclonal antibody technique, OX-LDL-EO6, LFL-DLH 3, OX-LDL-4E6 sandwich ELISA	In vivo	CVD endpoint predicted by OX-LDL; its level also indicates MI. In vivo OX-LDL link to atherosclerosis and its level in CVD individuals is more	Specificity of OXLDL is questionable	[113, 136–139]
Myeloperoxidase	Cohort study, case-control	Inflammatory neutrophil and basophil activate MPO, MAPK/NF-*κ*B signaling	Peroxidation assay, spectrophotometrically, ELISA	In vivo and in vitro	MPO linked to acute MI, CAD	Specific	[71, 140–143]
